# Pharmacist resource use across the United States: implications for teaching and practice

**DOI:** 10.5195/jmla.2026.2424

**Published:** 2026-10-01

**Authors:** Rachel Whitney, Hilary Jasmin, Christopher Alan Giuliano, Audrey Banner Kostrzewa, Melissa Hunter, Christopher S. Wisniewski, Emily F. Gorman

**Affiliations:** 1 whitnera@musc.edu, Research & Education Informationist, MUSC Libraries, Medical University of South Carolina, Charleston, SC; 2 jasmin@ohsu.edu, Information Specialist, Center for Evidence-based Policy, Oregon Health & Science University, Portland, OR; 3 ek2397@wayne.edu, Professor (Clinical), Department of Pharmacy Practice, Eugene Applebaum College of Pharmacy and Health Sciences, Wayne State University, Detroit, MI; 4 audrey.kostrzewa@cuw.edu, Professor, Department of Pharmacy Practice, Concordia University Wisconsin School of Pharmacy, Mequon, WI; 5 mhunter2@uwyo.edu, Director, Drug Information Center, School of Pharmacy, University of Wyoming, Laramie, WY; 6 wisniews@musc.edu, Professor, Department of Clinical Pharmacy and Outcomes Sciences, Medical University of South Carolina, Charleston, SC; 7 efgorman@hshsl.umaryland.edu, Research and Education Librarian, School of Pharmacy Librarian, Health Sciences and Human Services Library, University of Maryland, Baltimore, Baltimore, MD

**Keywords:** Drug information, electronic resources, pharmacy education, pharmacy practice, collection development

## Abstract

**Objective:**

To analyze practicing pharmacists' use of drug information (DI) resources in the United States.

**Methods:**

An electronic survey was distributed to practicing US pharmacists through professional organization email lists and social media. The survey questionnaire asked respondents which subscription-based DI resources are provided through their employer, whether they personally pay for additional resources, how often they use subscription-based resources, and other related questions.

**Results:**

The survey received 430 responses, 387 of which were able to be fully analyzed. The resources that respondents most frequently had access to through their employer were UpToDate (77.0%), Lexicomp (74.9%), and Micromedex (62.3%). Many respondents (40.8%) paid for subscriptions with personal funds, and 28.9% reported never experiencing access issues due to payment barriers. Respondents in community settings were more likely to have paid for resources with personal funds (57.1%, p<0.05) and have less access to resources (51.4%, p<0.05). In the open-text responses, the most desired resources were point-of-care tools (UpToDate) and DI databases (Lexicomp).

**Conclusions:**

US pharmacists experience a varied level of access to DI resources through their employer. Pharmacy educators should focus their instruction on resources that are the most accessible to practicing pharmacists. Many, especially community pharmacists, pay out of pocket for desired resources, presenting an opportunity to advocate for increased resources.

## INTRODUCTION

The utilization of drug information (DI) is a crucial aspect of pharmacy practice. Pharmacists are asked to respond to a variety of DI questions every day, most of which directly impact patient care. Although these questions vary, responses are generally formulated through a systematic approach, typically taught in pharmacy schools by both pharmacists and librarians. In brief, the systematic approach involves gathering appropriate background information to formulate the appropriate question(s), systematically searching the literature to gather information, analyzing and synthesizing a response, disseminating the information, and following up [1]. Central to carrying out this process is access to and familiarity with using DI resources.

Exposure to DI resources often begins during pharmacy education, taught by both pharmacists and librarians. Librarians contribute to this process by teaching research skills and introducing students to drug information databases, point-of-care tools, and larger compendia that support their learning [2]. However, the depth and extent of pharmacy trainees’ exposure to these licensed information tools is contingent upon institutional access to them. Existing studies have researched pharmacists’ preference for particular resources but few have examined the actual availability of specific resources in their practice settings [3–5]. Those studies that have examined resource availability are either not representative of current practice or are restricted to a local area (e.g. one US state) [6–10]. So although pharmacists’ value within the health care system is well established, less is known about whether they are consistently provided with the resources required to practice at their full potential.

Understanding which resources pharmacists currently use in practice is important for several reasons. The primary purpose of this study was to identify resources that can guide DI instruction in pharmacy schools. Because time to educate in pharmacy curricula is limited, there is a need for educators to select the most relevant resources for their students; information that is generally not available in the literature. Aligning educational exposure with use can narrow the gap between training and practice, ensuring that new pharmacists are prepared to use the resources most likely to be accessible in their work settings. Therefore, the present study seeks to inform DI educators of the current state of subscription-based resource availability to practicing pharmacists across the United States (US), with a long term goal of equipping future pharmacists with the knowledge and skills to effectively utilize the resources they will most often encounter in practice.

## METHODS

### Survey

We conducted a cross-sectional anonymous survey of US practicing pharmacists from April to May of 2024. The primary purpose of the survey was to inquire about the use and availability of pharmacy resources in various practice settings. Secondary outcomes included evaluation of resource use depending on if the employer provided access to the resource, the amount paid by respondents for resources, and resource use by year of pharmacy school graduation.

To accomplish this the authors developed a survey consisting of 12 items, including questions on practice/employment setting, subscription-based resources provided through employers, payment for additional resources with personal funds, frequency of use of subscription-based resources, and other related questions (see supplementary files for full survey instrument). Of note, the resource named “Lexicomp” in the survey has since been renamed UpToDate Lexidrug. This paper is using the name Lexicomp because that is what the resource was called for the duration of the study. Three survey questions allowed qualitative responses: 1) Which of the following subscription/fee-based resources are provided to you through your organization/employer? 2) In your current or most recent job, what resources did you use that were unavailable to you in pharmacy school? 3) If you could have your employer provide access to one paid/subscription based resource that is currently unavailable to you, what would it be and why?

The survey was piloted with a small number of pharmacist colleagues of the authors to identify any items that needed clarification. Authors utilized their professional and social organizations and networks, as well as institutional peers, to disseminate the survey. The recruitment information was distributed through email lists and social media. A full distribution list is provided in the supplementary materials. No compensation was offered to participants, and no personally identifying information was collected. Data were collected and analyzed through Qualtrics, with additional analysis in SPSS vs. 29.0. This survey was approved as exempt research by the Concordia University Wisconsin Institutional Review Board.

### Quantitative and Qualitative Analysis

Data were described using the mean and standard deviation or median with interquartile range for continuous data depending on the distribution of the data. Ordinal data were described using median with interquartile range. Categorical data were described using frequencies and proportions. Student’s t-test was used to analyze differences between two means. The Mann-Whitney U test was used to assess differences between distributions of two independent groups when the data are not normally distributed or ordinal. The chi-square test was used to assess associations between categorical variables. Differences in responses were compared across practice setting and graduation year (which was collected in 10-year increments). A comparative analysis between pharmacists graduating prior to and after 2009, the year in which the PharmD degree became required, was conducted to explore potential differences with resource availability across larger groups. This date was selected for convenience and as an approximate cutoff for a generational shift in graduating pharmacists. Additionally, the relationship between access to resources and use of resources was evaluated. All data were analyzed using SPSS v. 29.0 and a predetermined alpha of 0.05 was used to detect statistical difference. A convenience sample of 100 was used as the minimum number of respondents.

For qualitative analysis, grounded theory (including line-by-line open coding and thematic analysis) was utilized by two independent reviewers for each of the three open-ended questions. Any disagreements were settled by a third reviewer. NVivo was used for assigning codes for thematic analysis.

## RESULTS

### Demographics

A total of 430 participants initiated the survey, with 10 participants being excluded for not being a practicing pharmacist. Of the remaining 420 participants, all completed the demographics section of the survey, 385 completed all questions, and 2 participants had partial completion of the resource use section. Participants that only completed the demographic section were more likely to be employed in an academic hospital pharmacy setting compared to other settings (9.8% vs 4.2%, p=0.03). There were no other differences in participants that completed the demographics section only. We excluded participants who did not complete the full survey from the remainder of the analysis. Of the 387 full and partial respondents, 49 US states were represented (excluding Hawaii), 48.6% graduated after 2009, and 58.7% practiced in hospital, 18.7% in ambulatory care, 9% in community, and 14% in “Other.” Forty-four percent of respondents either were a preceptor or were employed by a pharmacy school. Respondents in the “Other” category included government (2.1%), pharmaceutical industry (1%), and others that were provided via free text (10.3%).

### Quantitative Results

Overall responses are provided in Table 1. Many respondents paid for subscriptions with personal funds (40.8%), with a similar percentage noting they used resources that were unavailable in school (41.1%). Of those that personally paid for resources (n=158), the number paid for was most frequently between 1 and 3 resources (88.2%), with most (58.2%) paying between $101- $500 per year. Only 28.9% of respondents never had access issues related to cost over the course of the year. There were differences in response depending on graduation year. Respondents that graduated before 2009 more frequently paid for resources with personal funds, had more access to resources, paid a higher monetary amount for resources, and less frequently had access issues due to cost (Table 1).

**Table 1:** Responses by graduation year

		Overall n=387	Before 2009 n=196	2009 or After n=188
Paid subscriptions with personal funds		158 (40.8)	94 (48)*	64 (34)
Used any resources that were unavailable in school		159 (41.1)	99 (50.5)*	60 (31.9)
Access to subscription & fee-based resources relative to school	More Less Same	120 (31) 141 (36.4) 89 (23)	90 (45.9)* 43 (21.9) 32 (36.4)	30 (16) 98 (52.1) 56 (29.8)
Number of resources paid for (n=161)	1-3 4-6 >6	142 (88.2) 14 (8.7) 2 (1.2)	80 (85.1) 11 (11.7) 2 (2.1)	62 (92.5) 3 (4.5) 0 (0)
Paid per year amount (n=158)	$1-100 $101-500 $501-1,000 $1,001-5,000	36 (22.8) 92 (58.2) 27 (17.1) 2 (1.3)	14 (15.1)* 56 (60.2) 21 (22.6) 2 (2.2)	22 (33.8) 37 (56.9) 6 (9.2) 0 (0)
How often no access due to cost	Daily Several times per week Several times per month Several times per year Never	14 (3.6) 37 (9.6) 78 (20.2) 144 (37.2) 112 (28.9)	5 (2.6)* 19 (9.7) 37 (18.9) 67 (34.2) 68 (34.7)	9 (4.8) 18 (9.6) 41 (21.8) 77 (41) 43 (22.9)

Values described as n(%); *p<0.05; Questions related to number and amount of paid resources were limited to those who had answered that they paid for resources with personal funds; unsure responses not displayed in table. 2009 was used as the time frame because the PharmD became required in that year, and also represented an approximate cutoff for a generational shift in graduating pharmacists.

Questions related to number and amount of paid resources were limited to those who had answered that they paid for resources with personal funds; Unsure responses not displayed in table.

There were also differences in access to resources across practice sites. Respondents in the community setting were more likely to have paid for resources with personal funds, had less employer-provided access to resources, and paid a higher monetary amount for resources (Table 2). Specific employer-provided resources by site are provided in Table 3. The community pharmacy setting had on average a smaller number of resources provided by employers (2.60) compared to the hospital (6.50) and ambulatory care (6.96). The most frequently used resources in participants that had employer-provided access were Lexicomp (90.7%), UpToDate (79.2%), Micromedex (68%), and subscription journals (54.9%). The most frequently used resources in participants without employer-provided access were UpToDate (18.2%), Lexicomp (15.6%), Pharmacist's Letter (10.6%), and Sanford Guide (10.5%). Lastly, we conducted a post hoc analysis evaluating the academic setting (Table 2, 3). Respondents with an academic affiliation had more access to resources and were less likely to pay for resources with personal funds compared to those with no academic affiliation.

**Table 2:** Responses by practice site

		Hospital n=227	Ambulatory Care n=72	Community n=35	Academia n=178
Paid subscriptions with personal funds		88 (38.8)	24 (33.3)	20 (57.1)*	76 (34.5)^
Used any resources that were unavailable in school		85 (37.4)	30 (41.7)	13 (37.1)	96 (43.6)
Access to subscription & fee-based resources relative to school*	More Less Same	62 (27.3) 90 (39.6)* 49 (21.6)	22 (30.6) 23 (31.9) 24 (33.3)	10 (28.6) 18 (51.4)* 2 (5.7)	83 (37.7) 58 (26.4)^ 62 (28.2)
Number of resources paid for (n=161)	1-3 4-6 >6	78 (86.7) 8 (8.9) 2 (2.2)	21 (84) 2 (8) 1 (4)	19 (90.5) 1 (4.8) 0	66 (85.7) 9 (11.7) 1 (1.3)
Paid per year amount (n=158)	$1-100 $101-500 $501-1000 $1001-5000	27 (17.1)* 46 (51.7) 16 (18) 0	1 (4.2) 18 (75) 5 (20.8) 0	2 (10.5) 14 (73.7) 3 (11.1) 0	23 (30.3) 42 (55.3) 10 (13.2) 1(1.3)
How often no access due to cost	Daily Several times per week Several times per month Several times per year Never	10 (4.4) 20 (8.8) 54 (23.8) 83 (36.6) 60 (26.4)	3 (4.2) 5 (6.9) 14 (19.4) 31 (43.1) 19 (26.4)	1 (2.9) 7 (20) 4 (11.4) 10 (28.6) 13 (37.1)	14 (3.6) 16 (7.3) 26 (20.9) 86 (39.1) 65 (29.5)

Values described as n(%);

* p<0.05;

^ p<0.05 for academia vs no academia.

**Table 3:** Employer-provided resources by site

	Hospital n=227	Ambulatory Care n=72	Community n=35	Academia n=178
AccessPharmacy*	51 (22.5)	34 (47.2)	1 (2.9)	106 (48.2)^
APhA Pharmacy Library*	19 (8.4)	14 (19.4)	5 (14.3)	42 (19.1)^
ASHP eBooks	25 (11.0)	5 (6.9)	0 (0)	28 (12.7)
Clinical Key	50 (22.0)	15 (20.8)	0 (0)	66 (30)^
Clinical Pharmacology*	52 (22.9)	25 (34.7)	7 (20)	73 (33.2)^
Cochrane Library	72 (31.7)	31 (43.1)	0 (0)	102 (46.4)^
Dynamed	53 (23.3)	16 (22.2)	1 (2.9)	69 (31.4)^
Epocrates (paid)*	1 (0.4)	3 (4.2)	1 (2.9)	5 (2.3)
Facts & Comparisons*	41 (18.1)	22 (30.6)	16 (45.7)	67 (30.5)^
Lexicomp*	185 (81.5%)	55 (76.4)	11 (31.4)	190 (86.4)^
MedicineComplete	3 (1.3)	2 (2.8)	0 (0)	4 (1.8)
Micromedex*	160 (70.5)	47 (65.3)	2 (5.7)	159 (72.3)^
Natural Medicines	78 (34.4)	36 (50)	5 (14.3)	102 (46.4)^
Pharmacists Letter	114 (50.2)	35 (48.6)	12 (34.3)	108 (49.1)^
PharmaPendium	0 (0)	1 (1.4)	0 (0)	1 (0.5)
Pyrls	1 (0.4)	2 (2.8)	1 (2.9)	8 (3.6)
Reference books*	125 (55.1)	29 (40.3)	9 (25.7)	131 (59.5)
Sanford*	110 (48.5)	16 (22.2)	1 (2.9)	80 (36.4)
Subscription journals	87 (38.3)	33 (45.8)	5 (14.3)	120 (54.5)
Stat!Ref	28 (12.3)	12 (16.7)	0 (0)	40 (18.2)
UpToDate*	200 (88.1)	59 (81.9)	11 (31.4)	187 (85)
VisualDx	4 (1.8)	5 (6.9)	0 (0)	11 (5)

Values described as n(%);

* p<0.05’

^ p<0.05 for academia vs no academia.

Having employer-provided access to resources significantly increased use of those resources (Table 4). The notable finding in Table 4 is the number of resources that were used by people despite not being provided access through their employer. For example, 18% of the respondents whose employers did not provide access to UpToDate still regularly used this resource, which suggests the respondents pay out of pocket for a subscription.

**Table 4:** Relationship between employer-provided access of resources and regular use

	No Employer-Provided Access	Employer-Provided Access
Clinical Pharmacology*	2/281 (0.7)	45/105 (42.9)
Cochrane Library*	5/257 (1.9)	29/129 (33.4)
Dynamed*	1/297 (0.3)	28/89 (31.5)
Epocrates (paid)*	2/378 (0.5)	2/8 (25)
Factors & Comparisons*	2/292 (0.7)	39/94 (41.5)
Lexicomp*	15/96 (15.6)	263/290 (90.7)
MedicineComplete	0/381 (0)	0/5 (0)
Micromedex*	8/145 (5.5)	164/241 (68.0)
Natural Medicines*	9/245 (3.7)	56/141 (39.7)
Pharmacists Letter*	21/199 (10.6)	85/187 (45.5)
PharmaPendium	0/385 (0)	0/1 (0)
Pyrls*	12/375 (3.2)	6/11 (54.5)
Reference books*	12/194 (6.2)	59/192 (30.7)
Sanford*	26/247 (10.5)	75/139 (54)
Subscription journals*	16/233 (6.9)	84/153 (54.9)
Stat!Ref*	2/338 (0.6)	10/48 (20.8)
UpToDate*	16/88 (18.2)	236/298 (79.2)
VisualDx	1/374 (0.3)	2/12 (16.7)

Values described as n(%);

* p<0.05;

Values indicate numbers of regular users over number without or with access

### Qualitative results

Five resource-specific themes emerged in qualitative analysis in response to the first question of “Which of the following subscription/fee-based resources are provided to you through your organization/employer?”. These themes were tertiary compendia, journals/databases, professional association-related resources, specifically named resources including point-of-care tools, and nonspecific responses like “Google” or “Internet”. Additionally, two perception-specific themes emerged: “I have everything I need”, and “I don’t know what I might need”. These seven codes were applied to all open-text responses. Additionally, the heatmap below (Figure 1) illustrates the most popular resources mentioned and their quantities.

**Figure 1:**
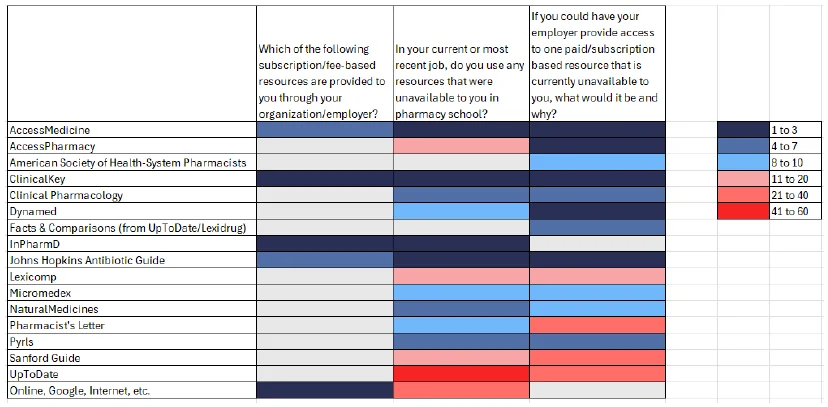
Heatmap of resources named in open-text responses in alphabetical order

*Increments in this map were made to differentiate less frequent responses from more frequently occurring responses, therefore the intervals are smaller for lower occurring responses.

Concerning the relationship between resource access and year of graduation, the quantitative results corresponded with the qualitative answers. In response to the question “In your current or most recent job, do you use any resources that were unavailable to you in pharmacy school?”, 15% (n=33) of qualitative responses noted a lack of access due to their age at the time they attended school, noting that they primarily used paper resources at the time:

“Almost all of the online resources were not available when I was in pharmacy school - except I would buy Lexicomp books.”

“I was in school pre-internet, so there are a LOT more available to me now. And they are electronic!”

“Online didn't exist when I was in pharmacy school so none of these were available - we used books only.”

In the final open-ended question, “If you could have your employer provide access to one paid/subscription based resource that is currently unavailable to you, what would it be and why?” responses had a dynamic range. More specific responses included access to American Society of Health-System Pharmacists and Pharmacist’s Letter, both mentioned for their continuing education (CE) resources. Journals listed by name included *The American Journal of Compounding Pharmacy*, *Annals of Pharmacotherapy*, *The American Journal of Health Systems Pharmacists*, *The International Journal of Pharmaceutical Compounding*, *The Journal of the American Medical Association*, *The Journal of Clinical Pharmacology*, *The Journal of Pediatric Pharmacotherapy*, and *New England Journal of Medicine*. There were also many broad responses, such as:

“Access to ebooks”

“Access to the most common journals would be helpful”

“Anything to help us get access to ALL journal articles (pubmed)”

“Better direct access to primary literature. Our library is good at tracking down articles, but I hate feeling like I'm badgering them with requests.”

## DISCUSSION

This study is the first to survey a national sample of pharmacists evaluating subscription-based resource use in practice. Surprisingly, a significant proportion of pharmacists purchased their own resources. Unsurprisingly, not having employer-provided access to specific resources significantly decreased their use. Furthermore, many pharmacists reported not having access to resources due to cost. These findings suggest that for some pharmacists, resource availability may be inadequate, which has the potential to impact patient care. For pharmacists in those situations, these data have the potential to help them advocate for access to additional tools necessary for optimal patient care.

While potentially inadequate access was identified across all settings, community access was lacking more than hospital or ambulatory care. This is especially problematic as community pharmacies impact a larger portion of the population comparatively, and are the most accessible to the public, often acting as a sole healthcare touch point for patients [11]. Identifying resources that are most frequently used in practice can help pharmacists advocate to their employers for resources that will be most useful. Additionally, it can help inform collection development budgets to align resources with those that are more likely to be used in practice.

These results have important implications for teaching DI in the pharmacy curriculum. Broader guidance is provided by the 2025 ACPE standards and the 2013 Entrustable Professional Activities; [12,13] however, specific topics and resources needed to meet this guidance are currently not standardized. As a result, students enter advanced rotations and clinical practice with widely varying experiences with resources such as drug information databases (e.g. Micromedex, UpToDate), literature databases (e.g. PubMed), and reference texts (e.g. *Drugs in Pregnancy and Lactation*).

The high usage reported for Lexicomp, UpToDate, Micromedex, and subscription journals indicates that these remain important resources on which to educate students. However, once students graduate, they are unlikely to have access to many of these resources. This suggests a need to introduce pharmacy trainees to a broader range of resources, including lower cost tools they will have access to as practitioners. Equipping students with foundational skills such as literature searching, critical appraisal, and reading drug monographs will prepare them to engage in lifelong learning and engage in information seeking in their workplace.

The wide variety in the types of paid/subscription resources that respondents want access to supports teaching students how to use the data that is presented on a broader level. For example, adverse drug reactions (ADRs) are presented in a variety of different manners depending on the resource [14]. Teaching students how to interpret presentation of ADRs, whether it be quantitative or qualitative, and considering whether comparator percentages are included in the resource would prepare students to interpret ADRs across a variety of sources. Lastly, access to free resources, such as those provided by government and professional organizations, should be presented in case subscription-based resource access is an issue.

In addition to teaching students how to utilize existing DI resources, the findings in this study underscore the importance of teaching students how to stay current with pharmacy resources overall. Respondents who attended pharmacy school prior to the creation and accessibility of electronic resources discussed solely being taught to use textbooks and print resources for DI while in pharmacy school because many of these core electronic resources either did not exist, or were restricted to print copies. However, this issue is not isolated to those who attended pharmacy school prior to the development of online DI resources. The availability of electronic resources has changed even over the last five years with the addition of new databases (e.g. FirstLine), modifications to existing databases (e.g. name changes of titles or databases), and dissolution or merging of resources (e.g. ToxNet and LactMed are no longer individual databases). Changes to electronic resources are likely to continue as technology advances and artificial intelligence becomes more prevalent. Pharmacy educators must embrace the challenge of teaching students how to utilize existing resources while also equipping them with the skills to use new or changed resources.

This study has limitations to consider. Although survey respondents included pharmacists working across a variety of settings, hospital practice was more represented compared to other settings, such as community practice. A response rate was not calculable due to the online distribution methods used for sharing the survey, and responses to the survey do have a potential for response bias; the survey did not explicitly ask respondents if they had no access to any type of DI resources, meaning that the sample may have underrepresented pharmacists with a complete lack of access to DI resources. Additionally, the survey did not ask whether respondents felt they had sufficient access to the resources they needed. Lastly, the survey did not capture usage of free drug information resources and how use of those tools may impact use of paid resources. Future research should compare the use of paid and free resources, as well as investigate whether free resources provide sufficient information for high-quality pharmacy practice.

## CONCLUSION

This nationwide survey found pharmacists frequently paid with personal funds to access subscription-based drug information resources. Disparities in access existed across specific practices, with community pharmacists more frequently paying for resources. Additionally, there was a strong preference for specific resources (e.g., UpToDate, Lexicomp), while other resources were used infrequently (e.g. PharmaPendium, Epocrates). The results of this survey can be used to inform teaching and resource allocation.

## Data Availability

Data associated with this article are available in OSF at https://osf.io/p7uz6/files/osfstorage.

## SUPPLEMENTAL FILES

**Appendix A:** Survey Distribution List

**Appendix B:** Survey
