# Supplementary material for: Pharmacist resource use across the United States: implications for teaching and practice
Source: J Med Libr Assoc. 2026 Oct 14;114(4):410–8. doi: 10.5195/jmla.2026.2424 (PMC13670230; doi:10.5195/jmla.2026.2424)
Supplement: Supplementary file 1 [file jmla-114-4-410-s01.docx]

# Survey distribution list

The survey was sent or posted to the following:

- American Association of Colleges of Pharmacy (AACP)
  - Pharmacy Practice Section
  - Drug Information and Library Science Section
  - Laboratory Instructors Special Interest Group
  - Women Faculty Special Interest Group
- American College of Clinical Pharmacists (ACCP)
  - Drug Information PRN
- American Pharmacists Association (APhA)
  - Open Forum
  - Pharmacy Section
- American Society of Health-System Pharmacy (ASHP)
- Facebook
  - Pharmacists Moms group
  - Peloton Pharmacists group
- GPO Vizient listserv
- LinkedIn
- Medical Library Association (MLA)
  - Pharmacy and Drug Information Caucus
- National Community Pharmacists Association (NCPA)
  - Association-wide newsletter
- Pharmlib listserv
- Wyoming Pharmacy Association
