# Supplementary material for: Pharmacist resource use across the United States: implications for teaching and practice
Source: J Med Libr Assoc. 2026 Oct 14;114(4):410–8. doi: 10.5195/jmla.2026.2424 (PMC13670230; doi:10.5195/jmla.2026.2424)
Supplement: Supplementary file 2 [file jmla-114-4-410-s02.docx]

Informed Consent

You are being asked to participate in this research study because you are a practicing pharmacist. Participation in this research is voluntary and includes only those who choose to take part. The purpose of this survey is to identify the availability of drug information resources to practicing pharmacists.

Participating in this study involves ﬁlling out an online survey in Qualtrics consisting of multiple choice and open-ended questions. This online survey should take approximately 10 minutes to complete and will close on May 17, 2024.

There is a risk of loss of conﬁdentiality with participation but your responses will not be linked to you. Responses are anonymous.

Submission data (IP addresses, browser, other personal identiﬁers) are not being collected. The results may be published, but no one will be able to identify you as a participant. There are no direct beneﬁts to you from participating in this survey.

However, your participation may help us learn more about the availability of drug information resources in pharmacy practice. This information may lead to changes in pharmacy school curriculum and recommendations for employers that may beneﬁt future students and pharmacists.

You may decide not to participate in the survey and may exit the survey at any time without penalty. You are free to decline to answer any questions you do not want to answer for any reason. If you have questions at any time about the survey or the procedures, you may contact the Principal Investigator, Audrey Kostrzewa, at [audrey.kostrzewa@cuw.edu](mailto:audrey.kostrzewa@cuw.edu) or the Concordia University Wisconsin-Ann Arbor IRB Administrator, Dr. Janessa Doucette, at [janessa.doucette@cuw.edu](mailto:janessa.doucette@cuw.edu) or 262-243-2721. By continuing to the next page of the survey, you are choosing to participate in the survey, which implies your understanding and willingness to participate.

Demographics

Are you or were you ever a practicing pharmacist?

 Yes No

In which U.S. state/territory do you currently work?

When did you graduate from pharmacy school?

 prior to 1970

 1970-1979

 1980-1989

 1990-1999

 2000-2009

 2010-2019

 2020-2023

Which of the following best describes your current professional setting? If you are clinical/practice faculty, please select

pharmacy school and your relevant practice setting. If you serve as a preceptor for pharmacy trainees, please select preceptor and your relevant practice setting.

 Pharmacy school

 Hospital pharmacy - academic or university-afﬁliated

 Hospital pharmacy - not academic or university-afﬁliated

 Ambulatory care pharmacy - academic or university-afﬁliated

 Ambulatory care pharmacy - not academic or university-afﬁliated Community pharmacy - chain

 Community pharmacy - independent

 Community pharmacy - online only/mail-order Pharmaceutical industry

 Government agency Preceptor

 Retired or not currently employed

Other - please specify

Block 2

To the best of your knowledge, which of the following subscription/fee-based resources are available to you through your organization/employer or your preceptor afﬁliation? Select all that apply.

 AccessPharmacy

 APhA Pharmacy Library ASHP eBooks

 ClinicalKey

 Clinical Pharmacology Cochrane Library

 Dynamed

 Epocrates (paid version)

 Facts and Comparisons eAnswers Lexicomp

 MedicinesComplete Micromedex

 Natural Medicines Pharmacist's Letter PharmaPendium Pyrls

 Reference books (e.g. Handbook of Nonprescription Drugs, Goodman & Gilman's, etc.) either in print or as ebooks

 Sanford Guide to Antimicrobial Therapy

 Scholarly journal articles from subscription journals (i.e. not Open Access) Stat!Ref

 UpToDate VisualDx

Other - please specify

Which of the following subscription/fee-based resources do you use regularly? Select all that apply.

 AccessPharmacy

 APhA Pharmacy Library

 ASHP eBooks ClinicalKey

 Clinical Pharmacology Cochrane Library

 Dynamed

 Epocrates (paid version)

 Facts and Comparisons eAnswers Lexicomp

 MedicinesComplete Micromedex

 Natural Medicines Pharmacist's Letter PharmaPendium Pyrls

 Reference books (e.g. Handbook of Nonprescription Drugs, Goodman & Gilman's, etc.) either in print or as ebooks

 Sanford Guide to Antimicrobial Therapy

 Scholarly journal articles from subscription journals (i.e. not Open Access) Stat!Ref

 UpToDate VisualDx

Other - please specify

Which of the following best describes your situation regarding access to subscription/fee-based resources?

 I feel I have access to more subscription/fee-based resources than when I was a pharmacy student.

 I feel I have access to fewer subscription/fee-based resources than when I was a pharmacy student.

 I feel I have access to the same amount of subscription/fee-based resources as when I was a pharmacy student.

 I don't know/can't remember how many subscription/fee-based resources I had when I was a pharmacy student.

In your current or most recent job, do you use any resources that were unavailable to you in pharmacy school?

Yes - please specify

 No

 Unsure

How often are you unable to access resources/articles/information related to your work/practice due to payment barriers?

 Daily

 Several times a week Several times a month Several times a year Never

Have you ever paid for any subscription resources (including journals) with personal funds because your employer does not provide them and you feel they are essential for your work?

Please do not include licensure/certiﬁcation-related fees, organizational dues, or other similar fees.

 Yes No

 Unsure

If you have paid for resources with personal funds, about how many resources do you have you paid for?

 1-3

 4-6

 7-10

 11 or more Unsure

About how much money do you pay/have you paid per year for these resources?

 $1 - $100 $101 - $500

 $501 - $1000 $1001 - $5000

 More than $5000

If you could have your employer provide access to one paid/subscription-based resource that is currently unavailable to you, what would it be and why?

[Powered by Qualtrics](https://www.qualtrics.com/powered-by-qualtrics/?utm_source=internal%2Binitiatives&utm_medium=survey%2Bpowered%2Bby%2Bqualtrics&utm_content=%7B~BrandID~%7D&utm_survey_id=%7B~SurveyID~%7D)
